# An observational cohort study evaluating adalimumab concentrations for predicting non-recapture of biochemical response after dose escalation in patients with Crohn’s disease experiencing secondary loss of response

**DOI:** 10.1093/jcag/gwag002

**Published:** 2026-02-18

**Authors:** Davide De Marco, Kevin McHugh, Sophie Plamondon, Louis-Charles Rioux, A Hillary Steinhart, Melissa Horvat, Waqqas Afif

**Affiliations:** Division of Gastroenterology and Hepatology, McGill University Health Centre, Montreal, Québec, H3H 1V6, Canada; AbbVie Corporation, St Laurent, Québec, H4S 1Z1, Canada; Division of Gastroenterology, Université de Sherbrooke et Centre de Recherche Étienne Lebel, Sherbrooke, Québec, J1G 2E8, Canada; Gastroenterology Unit, Hôpital Maisonneuve-Rosemont, Montreal, Québec, H1T 2M4, Canada; Division of Gastroenterology and Hepatology, Mount Sinai Hospital & University of Toronto, Toronto, Ontario, M5G 1X5, Canada; AbbVie Corporation, St Laurent, Québec, H4S 1Z1, Canada; Division of Gastroenterology and Hepatology, McGill University Health Centre, Montreal, Québec, H3H 1V6, Canada

**Keywords:** Crohn’s disease, inflammatory bowel disease, biologics (IBD), inflammation

## Abstract

**Background:**

There is limited evidence to support optimal concentrations for therapeutic drug monitoring in patients with Crohn’s disease (CD) who experience a loss of response (LOR) to tumour necrosis factor antagonists. This study aimed to determine the threshold trough adalimumab concentration beyond which patients with LOR are unable to recapture a response after dose escalation.

**Methods:**

Enrolled patients had responded to adalimumab therapy after ≥16 weeks and subsequently experienced a secondary LOR, defined as a C-reactive protein (CRP) level ≥5 mg/L and/or a fecal calprotectin (FC) level ≥250 µg/g. Dosing was escalated from biweekly to weekly. Patients were then assessed for 12 weeks to determine their ability to recapture a response, defined as a CRP response (≥50% CRP decrease and/or CRP <5 mg/L) and/or FC response (≥50% FC decrease and/or FC <150 µg/g). The relationship between baseline trough concentration and non-recapture of biochemical response was evaluated using logistic regression.

**Results:**

Of 97 enrolled patients, 49 (50.5%) did not recapture a response to adalimumab after dose escalation. Baseline trough concentration was not associated with non-recapture of biochemical response (odds ratio, 0.98; 95% CI: 0.91-1.06; *P *= .626). There was no threshold trough concentration identified that was predictive of non-recapture of biochemical response. No new adalimumab safety signals were identified.

**Conclusions:**

No association was observed between trough adalimumab concentration and non-recapture of biochemical response after dose escalation for secondary LOR in CD. The threshold concentration above which dose escalation is ineffective remains unclear. ClinicalTrials.gov identifier: NCT02896985.

## Introduction

Tumour necrosis factor (TNF) antagonists are effective for the treatment of Crohn’s disease (CD). However, within 1 year of responding to these therapies, approximately 20%-46% of patients will have a confirmed secondary loss of response (LOR),^1-3^ defined as the return of active disease according to objective measures such as C-reactive protein (CRP) and fecal calprotectin (FC) levels.^4^ Several mechanisms may underlie LOR to TNF antagonists, including the presence of antidrug antibodies (ADAs), low drug concentrations, and pharmacodynamic failure due to non–TNF-mediated inflammatory pathways that may be driving the disease process (immune escape).^5-7^ Therapeutic options for patients with ADAs who have experienced an LOR include switching to other TNF antagonist therapies,^4^ while patients with low drug concentrations typically respond to dose intensification of TNF antagonist therapy in the absence or minimal presence of ADAs.^5^ Patients who are experiencing pharmacodynamic failure of TNF antagonist therapy despite higher drug concentrations are unlikely to respond to dose intensification or a different TNF antagonist and may derive greater benefit from switching to a drug with a different mechanism of action.^5^^,^^7^^,^^8^

Therapeutic drug monitoring (TDM) of TNF antagonist therapy has been proposed as part of the standard management of LOR to facilitate objective, evidence-based treatment decisions.^9-16^ Higher trough drug concentrations and dosing have been associated with better remission and response outcomes in several clinical trials of patients with inflammatory bowel disease (IBD) who received TNF antagonist therapy. Moreover, trough concentrations increase over time with dose escalation.^5^^,^^17^^,^^18^ However, the clinical utility of TDM remains limited by the varied optimal drug concentration ranges that have been suggested to predict treatment outcomes.^8^^,^^13^^,^^19^ Specifically, evidence is needed to determine drug concentration thresholds above which dose escalation provides no further benefit. Such thresholds could be used to prevent unnecessary exposure to ineffective therapy. Therefore, this study aimed to determine an upper trough adalimumab concentration that may predict the inability to recapture a response after dose escalation.

## Methods

### Study design

This post-marketing, multicentre, observational, prospective cohort study recruited patients with CD who had experienced a secondary LOR to adalimumab at 22 Canadian sites (ClinicalTrials.gov identifier: NCT02896985). The use of TDM of trough adalimumab levels at the time of LOR was followed by an increase from biweekly to weekly dosing. Patients were then assessed for 12 weeks after LOR to determine whether dose escalation led to the recapture of their response to adalimumab. Ethics approval was obtained from each individual participating institution, and all patients provided written informed consent to participate in the study.

### Patients

Study sites enrolled adults with CD who were 18 years of age or older and had an investigator-determined response to adalimumab after ≥16 weeks with doses of 160 mg at baseline, 80 mg at week 2, and 40 mg every other week thereafter. At baseline, enrolled patients had a secondary LOR, defined as active inflammatory disease evidenced by an elevated CRP level (≥5 mg/L) and/or an elevated FC level (≥250 µg/g). Patients who met the following key criteria were excluded from the study: primary non-response to adalimumab after ≥16 weeks; serious underlying disease other than CD; perianal or abdominal abscess; history of alcohol or drug abuse; a positive test for Clostridium difficile; pregnant or lactating; and use of any other investigational drug within 16 weeks of adalimumab therapy.

### Study procedures

The patients’ medical, surgical, and disease history; CRP, albumin, and FC concentrations; and their 2-item patient-reported outcome (PRO2) and Harvey-Bradshaw index (HBI) scores were collected in electronic case report forms. These data were obtained from hospital records, clinical charts, checklists, and patient-reported outcomes questionnaires. The FC levels were obtained from at-home or in-clinic samples measured using Dynacare kits and were reported to sites by an external laboratory. For TDM, serum trough adalimumab concentrations were measured by the bioanalysis department at AbbVie (Saint-Laurent, Québec, Canada) and analyzed using a central laboratory drug-sensitive ELISA test to determine whether these concentrations were predictive of the inability to recapture a response after dose escalation for secondary LOR. The 3 possible outcomes and therapeutic options based on the ELISA results were as follows: (1) Patients with undetectable or detectable drug concentrations and negative/low titre ADAs were treated with dose escalation; (2) patients with undetectable drug concentration and positive/high titre ADAs (defined as >100 AU/mL) were sensitized and switched to another TNF antagonist; and (3) patients with detectable drug concentrations, but in which ADAs could not be assessed due to limitations of ELISA testing, to determine the upper threshold of drug at which escalation would be ineffective.

Several efforts were made to address the potential for bias during cohort identification and laboratory measurements and processes in this study. Cohort eligibility was predefined in the entry criteria, and the use of a central laboratory with training of physicians and site personnel helped to ensure the consistency of TDM. In addition, a monitoring plan was designed specifically for quality control in this study.

### Outcomes

The primary endpoints of this study were the proportion of patients who did not recapture a response after dose escalation and the relationship between baseline trough adalimumab concentration and the non-recapture of biochemical response. Recapture of biochemical response was defined as the recapture of a CRP and/or FC response. Recapture of a CRP response was defined as a ≥ 50% decrease in CRP level and/or CRP normalization (CRP <5 mg/L) for patients with an elevated CRP level at baseline. Recapture of an FC response was defined as a ≥ 50% decrease in FC level and/or FC normalization (FC <150 μg/g) for patients with an elevated FC level at baseline. For patients with elevations in both CRP and FC levels at baseline, those who recaptured either a CRP response or an FC response were considered to have recaptured a response. Proportions of patients who did not recapture a CRP response and of those who did not recapture an FC response were also evaluated separately as part of the primary analysis, as were relationships between these endpoints and baseline drug concentration.

Secondary endpoints were exploratory and included the relationship between baseline trough adalimumab concentration and the non-recapture of a response, adjusting for possible associations with sex and baseline disease severity, body mass index (BMI), immunomodulator use, corticosteroid use, albumin and haemoglobin levels, and antibody status; the relationship between baseline drug concentration and clinical outcomes at week 12 including clinical remission (defined as a PRO2 score of <8), CRP or FC normalization, ≥50% decrease in CRP or FC level, and concomitant corticosteroid initiation; and relationships between changes in CD markers (CRP, FC, PRO2, and HBI) and both baseline and final drug concentrations. A post hoc analysis was also conducted to evaluate the proportions of patients who did not recapture response after dose escalation in subgroups defined according to median baseline values including disease duration (≤5 and >5 years), BMI (≤25 and >25 kg/m^2^), albumin level (≤33 and >33 g/L), CRP level (≤4.4 and >4.4 mg/L), FC level (≤600 and >600 µg/g), PRO2 score (≤9 and >9), and HBI score (≤5 and >5); and according to baseline smoking status (smoker and non-smoker), CD localization (ileal, colonic, and ileo-colonic disease), and immunomodulator use (yes and no).

The safety of adalimumab was reviewed through the collection of solicited reports of serious adverse events (SAEs), malignancy among patients ≤30 years of age, adverse event (AE)-related discontinuations, unusual failures in efficacy, and pregnancy. Spontaneous reports of non-serious AEs were also reviewed.

### Statistical methods

The cohort of patients who received at least 1 dose of adalimumab after LOR was used for all statistical analyses. The proportions of patients with non-recapture of biochemical response, non-recapture of CRP response, and non-recapture of FC response (and corresponding 95% CIs) were calculated overall and for subgroups defined according to baseline factors. Univiariable logistic regression was used to evaluate relationships between baseline trough adalimumab concentration (independent variable) and the dependent variables of non-recapture of biochemical response, non-recapture of CRP response, non-recapture of FC response, and clinical outcomes. For clinical response and remission outcomes, patients who withdrew from the study due to treatment failure were considered non-responders and non-remitters, respectively. For the overall proportions, the odds ratio (OR) and its 95% CI, *P-*value, and the area under the receiver operating characteristic curve (AUC) and its 95% CI were determined. If there was a statistically significant association between trough concentration and non-recapture of biochemical response, the optimal threshold trough drug concentrations were obtained using the maximum Youden’s *J* statistic (*J* = sensitivity + specificity − 1), and a receiver operating characteristic curve was plotted. Thresholds were not determined if the 95% CI for the AUC value contained 0.5. Multivariate models for the relationships between baseline drug concentration and non-recapture of biochemical response, non-recapture of CRP response, and non-recapture of FC response that adjusted for possible associations with baseline factors were created by backwards selection, with *P *< .10 as the threshold. The full models included the following covariates: trough adalimumab concentration (forced into all final models), ADA positive status, sex, baseline immunomodulator therapy use, baseline corticosteroid use, prior TNF antagonist use, baseline BMI, and baseline albumin concentration (aOR, Table S4). Pearson and Spearman rank correlation coefficients were used to evaluate relationships between CD markers and both baseline and final trough adalimumab concentrations.

For the primary analysis, patients who did not complete the study (final study visit was not within 84 ± 7 days from baseline) due to treatment failure were classified as having non-recapture of a response; otherwise, patients who did not complete the study were treated as missing. Patients with both CRP and FC markers elevated at baseline who completed the study but with available data for only 1 marker that did not show a ≥50% decrease or normalization were considered to have non-recapture of biochemical response, whereas patients with one of the 2 markers elevated at baseline that was missing at the end of treatment were treated as missing. There was no imputation of missing data.

### Study size determination

A sample size of 90 patients was determined to provide a 92.8% probability of maintaining a 1-sided 95% lower confidence bound >0.70 for the AUC, assuming a true AUC of 0.85 and a 70% rate of recaptured response.

## Results

### Patients

**Figure 1:**
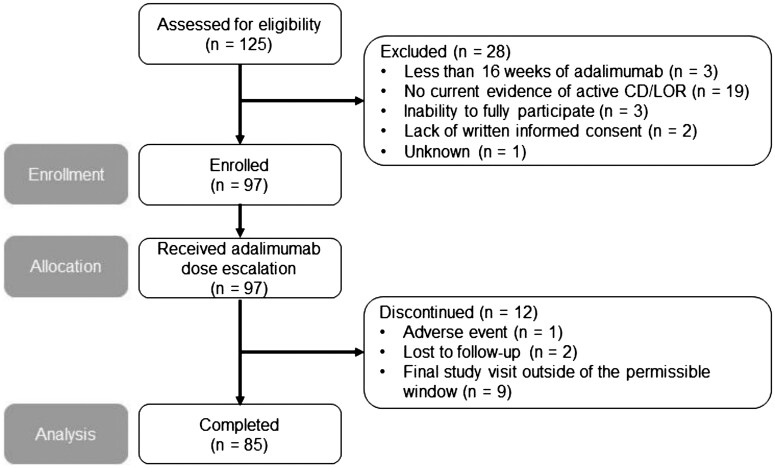
Patient disposition. CD, Crohn’s disease; LOR, loss of response.

A total of 125 patients with CD were assessed for eligibility, and 97 patients were enrolled and received escalated dosing of adalimumab treatment (Figure 1). Of the enrolled patients, 12 (12.4%) discontinued the study including 1 patient (1.0%) who discontinued because of an adverse event, 2 patients (2.1%) who were lost to follow-up, and 9 patients (9.3%) who had their final study visit outside of the permissible window (84 ± 7 days from baseline). Demographics and baseline characteristics in this study are shown in Table 1, and Table S1 shows demographic and baseline information stratified by response status. Among the 97 enrolled patients (mean age, 42.5 years), 49 (50.5%) were men, 25 (25.8%) had previously used TNF antagonist therapy, and 4 (4.1%) had ADAs at a mean concentration of 35.7 ng/mL. At baseline, the mean BMI was 26.3 kg/m^2^, mean disease duration was 12.1 years, mean disease marker levels were 10.8 mg/L for CRP and 1177.7 µg/g for FC, and the mean trough adalimumab concentration was 6.0 µg/mL.

**Table 1:** Demographics and baseline characteristics.

Characteristic	Patients (*n* = 97)
**Male, No. (%)**	49 (50.5)
**Age (years), mean ± SD**	42.5 ± 15.7^a^
**BMI (kg/m^2^), mean ± SD**	26.3 ± 5.8^b^
**Race, No. (%)**	
** White**	87 (89.7)
** Asian**	4 (4.1)
** Black**	3 (3.1)
** Native American**	1 (1.0)
** Unknown/not reported**	2 (2.1)
**Non-Hispanic/Latino ethnicity, No. (%)**	66 (68.0)
**Current/former tobacco use, No. (%)**	31 (32.0)
**Current/former alcohol use, No. (%)**	69 (71.1)
** <2 drinks per day**	61 (62.9)
** 2-4 drinks per day**	4 (4.1)
** >4 drinks per day**	4 (4.1)
**CD duration (years), mean ± SD**	12.1 ± 11.2^a^
**Disease location, No. (%)**	
** Small bowel**	35 (36.1)
** Colon**	24 (24.7)
** Colon and small bowel**	29 (29.9)
** Multiple locations/other**	9 (9.3)
**Current fistula presence, No. (%)**	12 (12.4)
** Internal location**	4 (4.1)
** Perianal location**	6 (6.2)
** Other location**	2 (2.1)
**PRO2 score, mean ± SD**	16.1 ± 19.7^c^
**HBI score, mean ± SD**	5.4 ± 4.4^c^
**Prior TNF antagonist use, No. (%)**	25 (25.8)
**Baseline corticosteroid use, No. (%)**	9 (9.3)
**Baseline immunomodulator use, No. (%)**	86 (88.7)
**C-reactive protein (mg/L), mean ± SD**	10.8 ± 17.9
**Fecal calprotectin (µg/g), mean ± SD**	1177.7 ± 1390.9^c^
**Albumin (g/L), mean ± SD**	40.8 ± 4.4^d^
**Baseline trough adalimumab concentration (µg/mL), mean ± SD**	6.0 ± 4.0^e^
**ADAs, No. (%)**	4 (4.1)

Abbreviations: ADAs, anti-drug antibodies; BMI, body mass index; CD, Crohn’s disease; HBI, Harvey-Bradshaw index; PRO2, 2-item patient-reported outcome; TNF, tumour necrosis factor.

a *n* = 95,

b *n* = 87,

c *n* = 93,

d *n* = 79,

e *n* = 86.

### Non-responders to dose escalation

**Figure 2:**
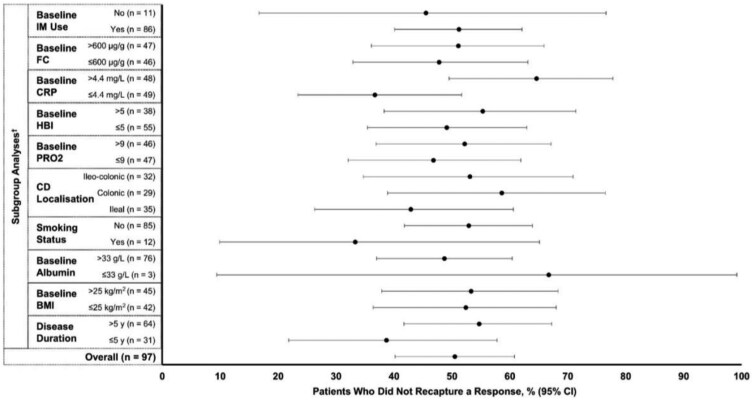
Proportions of patients with non-recapture of a response. BMI, body mass index; CD, Crohn’s disease; CRP, C-reactive protein; FC, fecal calprotectin; HBI, Harvey-Bradshaw index; IM, immunomodulator; PRO2, 2-item patient-reported outcome. ^†^Patients with missing subgroup classifiers were excluded from the relevant analyses.

At 12 weeks after adalimumab dose escalation for secondary LOR, 49/97 (50.5%) patients were unable to recapture a response (Figure 2). There were 29/47 (61.7%) patients with non-recapture of a CRP response (Figure S1) and 28/84 (33.3%) patients with non-recapture of an FC response (Figure S2).

Among several subgroups, the highest rates of non-response to dose escalation included the rate observed in patients with a baseline CRP level >4.4 mg/L (64.6%; 31/48) and the lowest non-response rates included the rate observed in the subgroup with a baseline CRP level ≤4.4 mg/L (36.7%; 18/49) (Figure 2). Subgroups with a baseline HBI score ≤5 (78.3%; 18/23) and a baseline PRO2 score ≤9 (76.5%; 13/17) had the highest rates of CRP non-response (Figure S1), and subgroups with colonic disease (44.0%; 11/25) and those with a baseline BMI ≤25 kg/m^2^ were among those with the highest rates of FC non-response (41.0%; 16/39) (Figure S2).

### Relationships between adalimumab concentration and non-response to dose escalation

**Figure 3:**
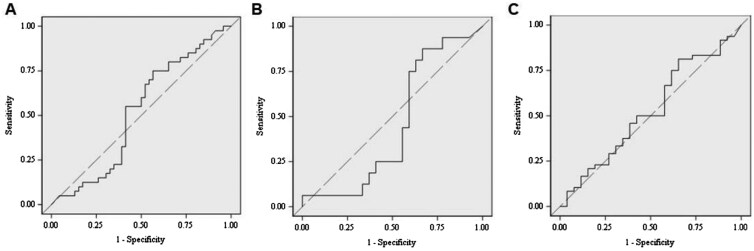
Relationships between baseline trough adalimumab concentration and non-recapture of biochemical response (A), a CRP response (B), and an FC response (C). CRP, C-reactive protein; FC, fecal calprotectin.

The baseline trough adalimumab concentration was not associated with the inability to recapture a response (Table 2, Figure 3A), a CRP response (Table 2, Figure 3B), or an FC response (Table 2, Figure 3C). Mean changes in trough adalimumab concentration from baseline to week 12 were also not associated with the inability to recapture a response (OR, 0.97; 95% CI: 0.91-1.03; *P *= .294), a CRP response (OR, 0.91; 95% CI: 0.83-1.00; *P *= .057), or an FC response (OR, 0.95; 95% CI: 0.87-1.02; *P *= .162). The mean ± SD increase in trough drug concentration from baseline to week 12 was similar among responders (7.10 ± 5.43 µg/mL) and non-responders (6.22 ± 4.85 µg/mL), CRP responders (6.97 ± 5.31) and CRP non-responders (4.63 ± 5.17), and similar among FC responders (7.64 ± 5.73 µg/mL) and FC non-responders (6.22 ± 4.88 µg/mL).

**Table 2:** Relationship between baseline trough adalimumab concentration and non-recapture of a response, CRP response, or FC response.

Outcome	Non-recapture of biochemical response	Non-recapture of CRP response	Non-recapture of FC response
**No. (%)**	49 (50.5)	29 (61.7)	28 (33.3)
**OR (95% CI) per 1 µg/mL baseline trough concentration**			
** Unadjusted analysis**	0.98 (0.91-1.06)	1.00 (0.91-1.10)	1.01 (0.92-1.10)
***P*-value**	.626	.954	.876
** Adjusted analysis^a^**	0.95 (0.83-1.08)	1.07 (0.90-1.27)	0.97 (0.84-1.12)
***P*-value**	.437	.457	.689
**AUC (95% CI)**	0.518 (0.430-0.606)	0.451 (0.326-0.576)	0.526 (0.427-0.626)

Abbreviations: ADA, anti-drug antibody; AUC, area under the receiver operating characteristic curve; BMI, body mass index; CRP, C-reactive protein; FC, fecal calprotectin; OR, odds ratio; TNF, tumour necrosis factor.

a The final model was created by backwards selection (*P *< .10 threshold); full model covariates: baseline trough adalimumab concentration (forced into all final models), ADA positive, sex, baseline immunomodulator therapy use, baseline corticosteroid use, prior TNF antagonist use, baseline BMI, and baseline albumin concentration. All final models included baseline trough adalimumab concentration, and only the relationship with non-recapture of CRP response also included baseline immunomodulator therapy use in the final model.

Threshold trough adalimumab concentrations predictive of non-recapture of biochemical response, CRP response, or an FCP response were not determined due to 0.5 being contained in the 95% CIs for the AUC values (Table 2).

### Relationships between adalimumab concentration and clinical outcomes

No significant relationship was observed between baseline trough adalimumab concentration and the outcomes of clinical remission (OR, 1.00; 95% CI: 0.91-1.10; *P *= .973), CRP or FC normalization (OR, 1.05; 95% CI: 0.97-1.13; *P *= .249), or a ≥50% decrease in CRP or FC level (OR, 1.05; 95% CI: 0.97-1.13; *P *= .249) (Table S2).

### Relationships between adalimumab concentration and CD markers

Baseline trough adalimumab concentration was not significantly associated with changes in the CD markers evaluated including CRP and FC levels, PRO2, and HBI score (Table S3). Final trough adalimumab concentration was not significantly associated with changes in FC level, PRO2, or HBI score but had a small negative correlation with the change in CRP level (Pearson correlation, −0.256; 95% CI: −0.452 to −0.032) (Table S3).

### Safety and tolerability

There were no new adalimumab safety signals in the study. Eight patients (8.2%) reported at least one AE during the study. Two patients (2.1%) experienced 3 SAEs that were unrelated to the study drug and resolved, including one patient (1.0%) who experienced appendicitis and discontinued the study. There was a single case of CD relapse, which led to discontinuation of adalimumab, and a single pregnancy with elective abortion. No malignancies in patients ≤30 years of age were reported, and there were no deaths.

## Discussion

Patients with CD who have experienced LOR to TNF antagonist therapy may undergo TDM of drug concentrations and ADA levels to determine appropriate subsequent management strategies. However, some of these patients who already have higher drug concentrations may not respond to dose intensification due to pharmacodynamic failure of the drug.^5-7^ Alternatively, the potential presence of ADAs could lead to the ineffectiveness of dose escalation.^20^^,^^21^ Although dose escalation and higher drug concentrations of TNF antagonist therapy have been shown to correlate with better clinical outcomes,^5^^,^^17^^,^^18^ more evidence is needed to identify the upper drug concentration thresholds above which dose escalation yields no further therapeutic benefit. The continued lack of clarity regarding specific upper thresholds for use in TDM may lead to suboptimal outcomes for patients who receive dose escalation without the possibility of therapeutic benefit and for patients who do not receive dose escalation that would have otherwise been beneficial.

In the current study of patients with CD who had experienced a secondary LOR, the relationship between trough adalimumab concentration and the non-recapture of a response after dose escalation was explored. A potential trough adalimumab concentration threshold to predict this inability to recapture a response to the drug was also examined. Results suggest that trough adalimumab levels increase over time after dose escalation, with about 50% of patients able to recapture a response—consistent with previously reported response rates in other studies of biologic therapies in IBD.^22^^,^^23^ However, there was no relationship between baseline trough adalimumab concentration and the non-recapture of a response in the current study. Furthermore, results indicate that patients most likely or least likely to recapture a response to adalimumab cannot be accurately predicted using threshold trough drug concentrations at the time of LOR or after dose escalation.

Notably, 25% of patients in this study had prior TNF antagonist exposure. This group may be less likely to respond to subsequent therapy in the same class, which may have influenced the lack of observed association with trough concentration and recapture of biochemical response. At the time this study was conducted, use of sequential TNF antagonists was common practice. Interestingly, rates of non-recapture of biochemical response were not numerically higher in patients with prior TNF antagonist exposure at baseline. An analysis of this patient subgroup is limited by the small sample size and the lack of data on antibodies to the initial TNF antagonist therapy.

In contrast to the results observed in the current study, a recent paper by Little et al.^4^ described a retrospective, multicentre study involving 131 patients with a 9-year median duration of CD. That study also had lower immunomodulator use at baseline (50.4%) compared to 88.7% in the current study. The authors reported that CD remission was associated with adalimumab drug levels following dose-intensification, instead of at the time of secondary LOR, and concluded that adalimumab drug levels at secondary LOR did not predict response to dose-intensification in patients with CD.^24^ Similarly, while a slightly greater increase was seen in trough levels post dose-intensification among patients with recapture response, this association was found to not be statistically significant. The secondary LOR was defined after 14 weeks, which was slightly earlier than ≥16 weeks in the current study. Little et al. followed adalimumab drug levels for 6-12 months after dose intensification, which was a longer duration than the 12 weeks (3 months) used in the current study, which might suggest that a timeframe longer than 3 months could be needed for escalation therapy to recapture response or remission.

Despite prior evidence suggesting positive associations between TNF antagonist drug concentrations and rates of response and remission,^5^^,^^17^^,^^18^ findings from the current study did not reveal any further data to support an optimal threshold to use in patients with LOR. Some challenges with TDM may preclude the ability to define specific ranges of TNF antagonist drug concentrations that predict treatment outcomes of dose escalation for LOR. The drug levels measured in serum using TDM may be unable to accurately reflect the actual pharmacodynamic effects of the drug or the levels present in the intestinal tissues that are being targeted.^25^ Moreover, therapeutic targets of TDM are often based on large cohorts of patients with a specific condition.^26^ This generalized approach does not account for interindividual variations in disease phenotype, treatment outcome of interest, disease activity, inflammatory burden, drug absorption, drug clearance, ADA presence, or lean body weight.^13^^,^^15^^,^^25^^,^^26^ Such variables may have been confounding factors in the previous studies that suggested correlations between higher drug concentrations and greater efficacy.

Based on the challenges of a generalized approach to TDM, an individual patient-level approach may be appropriate for patients treated with TNF antagonists who experience a secondary LOR. The guidelines on TDM in IBD by the American Gastroenterological Association suggest that dose escalation for LOR should be considered in patients with trough drug concentrations below 5 µg/mL for infliximab, 7.5 µg/mL for adalimumab, and 20 µg/mL for certolizumab pegol, while concentrations above these thresholds may warrant switching therapy.^7^^,^^8^ However, studies supporting these thresholds were not specific to patients with secondary LOR. It was also noted that these thresholds could vary for patients with certain clinical characteristics, such as those who are asymptomatic but have ongoing endoscopic disease activity, and should not be considered as uniform targets for all patients.^8^ Alternative TDM approaches that account for individualized factors may improve treatment outcomes at the individual patient level.^15^^,^^26^

The influence of confounding individual patient factors on the ability to define optimal drug concentration ranges among patients who experience a secondary LOR may also support a shift away from strict adherence to TDM for biologics in IBD. In a recent systematic review and meta-analysis of 9 randomized controlled trials of TNF antagonist therapy in IBD, similar clinical remission rates were reported among patients who received proactive TDM-driven dose adjustments (38%) and those who received clinically driven dose adjustments (42%).^27^ This analysis included 4 double-blind studies and 2 unblinded studies of adults with CD or UC that revealed no clinical benefits of TDM-based dose escalation.^28-33^ In contrast, the unblinded PAILOT study of adalimumab in paediatric CD and 2 unblinded studies of infliximab in adults with IBD demonstrated better clinical efficacy with proactive TDM than with non–TDM-based approaches.^34-36^ Furthermore, data supporting guideline-recommended trough drug concentration thresholds are more robust for infliximab than for adalimumab,^7^^,^^8^ suggesting that TDM may have greater clinical utility for selected biologics such as those with more flexible dosing.

This study has some key strengths, including its prospective cohort study design and evaluation of a well-characterized patient population of those treated with adalimumab dose escalation after experiencing a LOR. There was a high study completion rate of 87.6% (85/97; Figure 1), suggesting only minimal impact of missing data on the results. The relevant limitations should also be acknowledged. First, the generalizability of this study is limited by the intended descriptive nature of the observational design and the evaluation of fewer than 100 patients. Second, the entry criteria did not define an upper limit of disease duration for enrolment, allowing for the inclusion of patients with long-term disease of up to 41 years and potentially representing a more difficult-to-treat study population overall. Third, the definition of LOR was based on inflammatory biomarkers only and excluded any symptom-based assessments, resulting in some patients who were only mildly symptomatic being considered as having experienced a LOR. Finally, assays for TDM may vary across different laboratories,^37^ suggesting that values reported in this study may not be indicative of those that would be observed elsewhere.

In conclusion, among patients with CD who experienced a secondary LOR after initially responding to adalimumab therapy, approximately half were unable to recapture a response after escalation to once weekly dosing. We recommend that such patients undergo dose escalation in the absence of high titre ADAs. In this study, there was no evident relationship between adalimumab trough concentration and the failure to recapture a response. The threshold concentration above which dose escalation is not indicated after LOR remains unclear. No new adalimumab safety signals were identified.

## Supplementary Material

gwag002_Supplementary_Data

## Data Availability

Data relevant to this study will be made available to other researchers upon reasonable request.
