# Supplementary material for: An observational cohort study evaluating adalimumab concentrations for predicting non-recapture of biochemical response after dose escalation in patients with Crohn’s disease experiencing secondary loss of response
Source: J Can Assoc Gastroenterol. 2026 Feb 18;9(3):153–61. doi: 10.1093/jcag/gwag002 (PMC13232514; doi:10.1093/jcag/gwag002)
Supplement: gwag002_Supplementary_Data [file gwag002_supplementary_data.zip › PRADA manuscript Supplementary material revised 10Dec2025 clean.docx]

**An Observational Cohort Study Evaluating Adalimumab Concentrations for Predicting Non-recapture of Response After Dose Escalation in Patients With Crohn’s Disease Experiencing Secondary Loss of Response**

**SUPPLEMENTARY MATERIALS**

**Supplementary Table 1. Demographics and baseline characteristics by response status**

| **Characteristic** | **Patients (N=97)** | |  |
| --- | --- | --- | --- |
|  | **Non-recapture of Response (N=49)** | **Recapture of Response (N=48)** | **P Value** |
| Male, No. (%) | 24 (49.0) | 25 (51.0) | .760 |
| Age (y), mean ± SD | 42.4 ± 16.8^a^ | 42.7 ± 14.8 | .915 |
| BMI (kg/m^2^), mean ± SD | 26.4 ± 6.1^b^ | 26.1± 5.5^c^ | .800 |
| Race, No. (%) |  |  | .674 |
| White | 44 (89.8) | 43 (87.8) |  |
| Asian | 3 (6.1) | 1 (2.0) |  |
| Black | 1 (2.0) | 2 (4.1) |  |
| Native American | 1 (2.0) | 0 (0.0) |  |
| Unknown/not reported | 1 (2.0) | 1 (2.0) |  |
| Non-Hispanic/Latino ethnicity, No. (%) | 34 (69.4) | 32 (65.3) | .774 |
| Current/former tobacco use, No. (%) | 4 (8.2) | 8 (16.3) | .467 |
| Current/former alcohol use, No. (%) | 25 (51.0) | 32 (65.3) | .256 |
| <2 drinks per day | 27 (55.1) | 34 (69.4) |  |
| 2-4 drinks per day | 3 (6.1) | 1 (2.0) |  |
| >4 drinks per day | 2 (4.1) | 2 (4.1) |  |
| CD duration (y), mean ± SD | 12.7 ± 11.2^a^ | 11.6 ± 11.3 | .631 |
| Disease location, No. (%) |  |  | .678 |
| Small bowel | 15 (30.6) | 20 (40.8) |  |
| Colon | 14 (28.6) | 10 (20.4) |  |
| Colon and small bowel | 15 (30.6) | 14 (28.6) |  |
| Multiple locations/other | 8 (16.2) | 1 (2.0) |  |
| Current fistula presence, No. (%) | 6 (12.2) | 6 (12.2) | .446 |
| Internal location | 2 (4.1) | 2 (4.1) |  |
| Perianal location | 4 (8.2) | 2 (4.1) |  |
| Other location | 2 (4.1) | 0 (0.0) |  |
| PRO2 score, mean ± SD | 20.0 ± 24.1^b^ | 12.2 ± 13.3^a^ | .055 |
| HBI score, mean ± SD | 5.5 ± 4.0^d^ | 5.4 ± 4.7^e^ | .874 |
| Prior TNF antagonist use, No. (%) | 11 (22.4) | 14 (28.6) | .449 |
| Baseline corticosteroid use, No. (%) | 5 (10.2) | 4 (8.2) | .751 |
| Baseline immunomodulator use, No. (%) | 44 (89.8) | 42 (85.7) | .721 |
| C-reactive protein (mg/L), mean ± SD | 15.6 ± 23.5 | 5.9 ± 6.7 | .007 |
| Fecal calprotectin (µg/g), mean ± SD | 1345.5 ± 1609.8^b^ | 1013.4 ± 1130.8^a^ | .252 |
| Albumin (g/L), mean ± SD | 40.7 ± 4.6^f^ | 41.0 ± 4.3^g^ | .797 |
| Baseline trough adalimumab concentration (µg/mL), mean ± SD | 6.1 ± 4.6^b^ | 5.8 ± 3.2^g^ | .781 |
| ADAs, No. (%) | 2 (4.1) | 2 (4.1) | .241 |

Abbreviations: ADAs, anti-drug antibodies; BMI, body mass index; CD, Crohn’s disease; HBI, Harvey-Bradshaw index; PRO2, two-item patient-reported outcome; TNF, tumor necrosis factor.
^a^N=47. ^b^N=87. ^c^N=41. ^d^ N=39. ^e^N=48. ^e^N=48. ^f^N=45. ^g^N=40.

**Supplementary Table 2. Relationship between baseline trough adalimumab concentration and clinical outcomes**

| **Outcome** |  | **Clinical Remission^†^** | **CRP/FC Normalization^‡^** | **Substantial Decrease in CRP/FC^§^** |  |
| --- | --- | --- | --- | --- | --- |
| No. (%) |  | 29 (31.2) | 53 (54.6) | 53 (54.6) |  |
|  |  |  |  |  |  |
| OR per 1 µg/mL baseline trough concentration |  |  |  |  |  |
| OR (95% CI) |  | 1.00 (0.91, 1.10) | 1.05 (0.97, 1.13) | 1.05 (0.97, 1.13) |  |
| *p*-value |  | 0.973 | 0.249 | 0.249 |  |
|  |  |  |  |  |  |
| AUC (95% CI) |  | 0.499  (0.401, 0.597) | 0.544  (0.456, 0.632) | 0.544  (0.456, 0.632) |  |
|  |  |  |  |  |  |
| Threshold trough adalimumab concentration (µg/mL) |  | N/A^¶^ | N/A^¶^ | N/A^¶^ |  |
| Youden’s J threshold |  | – | – | – |  |
| Sensitivity at Youden’s J threshold |  | – | – | – |  |
| Specificity at Youden’s J threshold |  | – | – | – |  |
| ^†^ Defined as a PRO2 score of <8 at week 12.  ^‡^ Defined as a CRP level of <5 mg/L or an FC level of <150 µg/g at week 12.  ^§^ Defined as a ≥50% decrease from baseline to week 12. ^¶^ Thresholds were not determined if the 95% CI for the AUC value contained 0.5. Abbreviations: AUC, area under the receiver operating characteristic curve; CRP, C-reactive protein; FC, faecal calprotectin; OR, odds ratio; PRO2, two-item patient-reported outcome; N/A, not applicable. | | | | | |

**Supplementary Table 3. Relationship between baseline/final trough adalimumab concentrations and CD markers**

| **CD Marker^†^** | **Correlations with Baseline Trough Adalimumab Concentrations^‡^** | | **Correlations with Final Trough Adalimumab Concentrations^§^** | |
| --- | --- | --- | --- | --- |
|  | **Pearson Correlation (95% CI)** | **Spearman Correlation (95% CI)** | **Pearson Correlation (95% CI)** | **Spearman Correlation (95% CI)** |
| CRP | –0.087 (–0.310, 0.147) | –0.119 (–0.339, 0.115) | –0.256 (–0.452, –0.032) | –0.119 (–0.334, 0.108) |
| FC | –0.022 (–0.250, 0.210) | –0.010 (–0.240, 0.220) | –0.099 (–0.320, 0.133) | –0.086 (–0.308, 0.146) |
| PRO2 | 0.128 (–0.098, 0.340) | 0.118 (–0.108, 0.331) | –0.020 (–0.238, 0.201) | 0.012 (–0.208, 0.231) |
| HBI | 0.037 (–0.184, 0.255) | 0.093 (–0.130, 0.306) | –0.021 (–0.238, 0.199) | 0.069 (–0.153, 0.282) |
| Abbreviations: CD, Crohn’s disease; CRP, C-reactive protein; FC, faecal calprotectin; HBI, Harvey-Bradshaw index; PRO2, two-item patient-reported outcome. ^†^ Correlations made using changes from baseline.  ^‡^ PRO2: n = 78; CRP: n = 73; FC: n = 73; HBI: n = 80.  ^§^ PRO2: n = 80; CRP: n = 77; FC: n = 74; HBI: n = 81. | | | | |

**Supplementary Figure 1. Proportions of patients with non-recapture of a CRP response^†^**


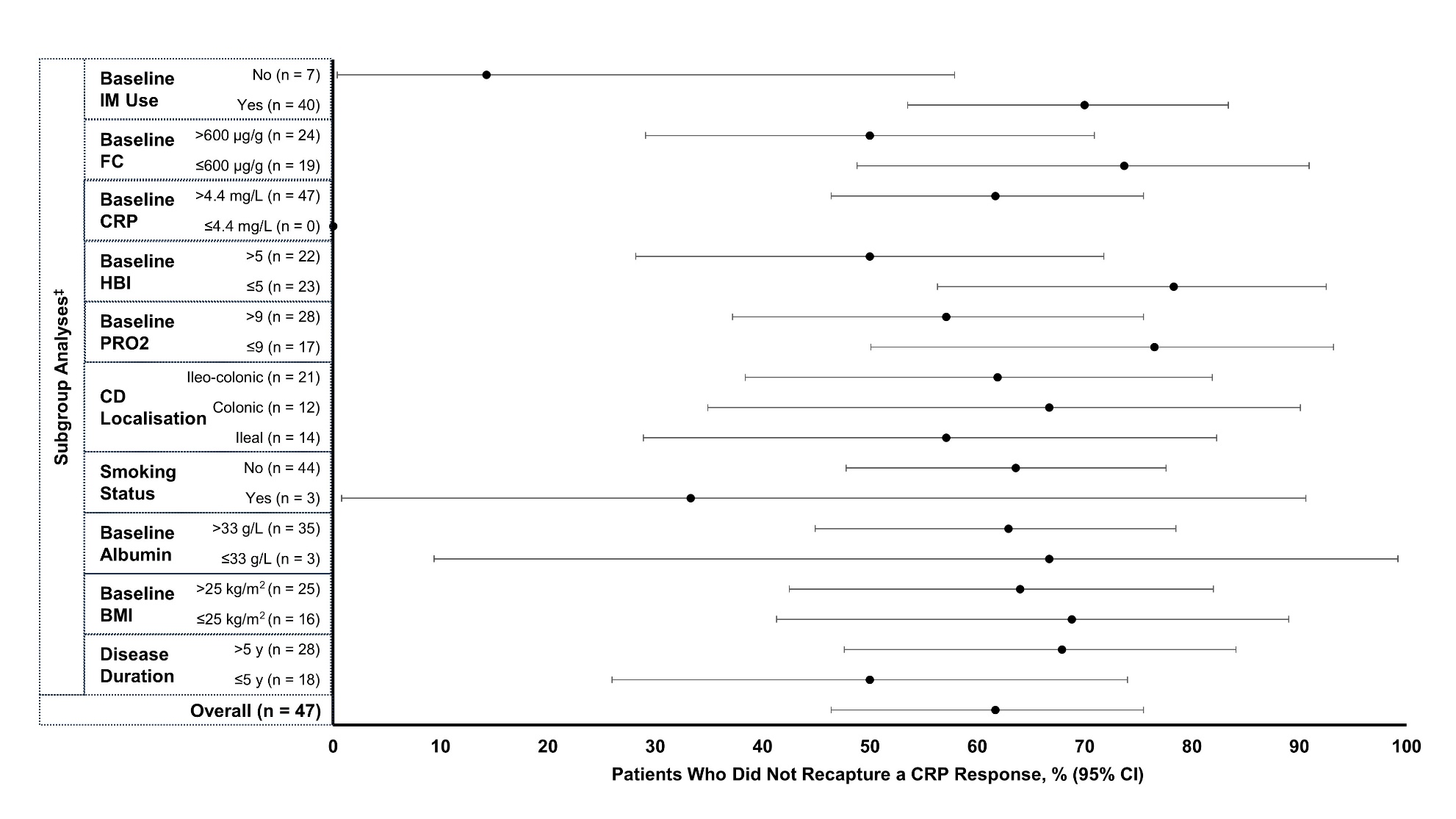


Abbreviations: BMI, body mass index; CD, Crohn’s disease; CRP, C-reactive protein; FC, faecal calprotectin; HBI, Harvey-Bradshaw index; IM, immunomodulator; PRO2, two-item patient-reported outcome.
^†^ Among patients with an elevated CRP level (≥5 mg/L) at baseline.
^‡^ Patients with missing subgroup classifiers were excluded from the relevant analyses.

**Supplementary Figure 2. Proportions of patients with non-recapture of an FC response^†^**


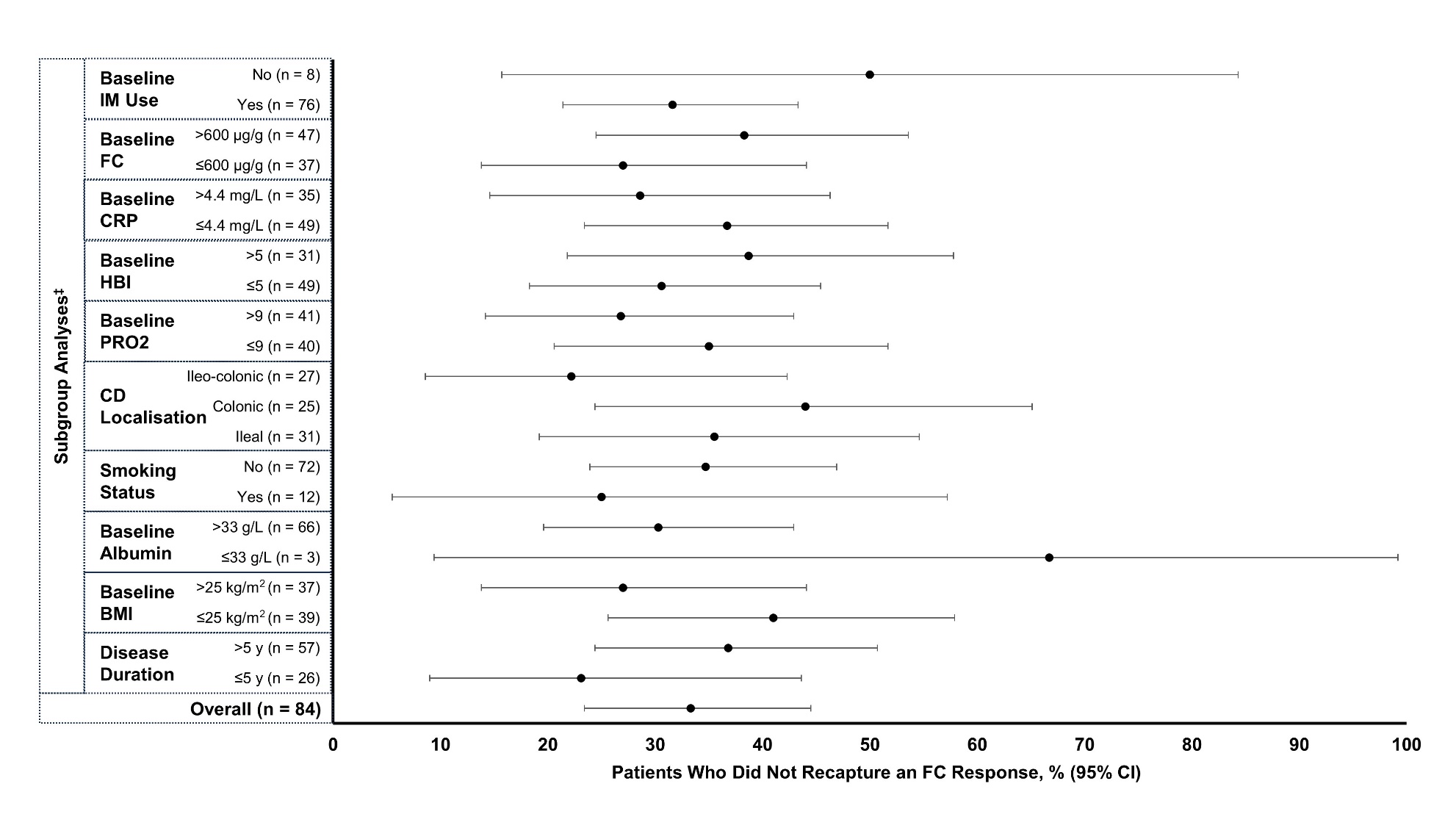


Abbreviations: BMI, body mass index; CD, Crohn’s disease; CRP, C-reactive protein; FC, faecal calprotectin; HBI, Harvey-Bradshaw index; IM, immunomodulator; PRO2, two-item patient-reported outcome.
^†^ Among patients with an elevated FC level (≥250 µg/g) at baseline.
^‡^ Patients with missing subgroup classifiers were excluded from the relevant analyses.

**Supplementary Table 4. Adjusted Odds Ratios in Multivariate Logistic Regression**

| **Covariate** | **aOR (95% CI)** | **p-Value** |
| --- | --- | --- |
| ADA trough | 1.05 (0.92 – 1.19) | 0.497 |
| Age | 1.00 (0.96 – 1.032) | 0.793 |
| Male Sex | 0.94 (0.33 – 2.66) | 0.413 |
| Baseline Immunomodulator Use | 2.01 (0.35 – 11.7) | 0.435 |
| Baseline Corticosteroid use | 0.41 (0.04 – 4.73) | 0.477 |
| Prior anti-TNF use | 0.84 (0.27 – 2.57) | 0.756 |
| Baseline albumin | 0.63 (0.86 – 1.09) | 0.629 |

Abbreviations: ADA, Adalimumab; TNF, Tumor Necrosis Factor
